# Long noncoding RNAs may impact podocytes and proximal tubule function through modulating miRNAs expression in Early Diabetic Kidney Disease of Type 2 Diabetes Mellitus patients

**DOI:** 10.7150/ijms.56551

**Published:** 2021-03-15

**Authors:** Ligia Petrica, Elena Hogea, Florica Gadalean, Adrian Vlad, Mihaela Vlad, Victor Dumitrascu, Silvia Velciov, Cristina Gluhovschi, Flaviu Bob, Sorin Ursoniu, Dragos Catalin Jianu, Petru Matusz, Agneta-Maria Pusztai, Andrei Motoc, Octavian M Cretu, Dana Radu, Oana Milas, Alina Golea-Secara, Anca Simulescu, Maria Mogos-Stefan, Mihaela Patruica, Lavinia Balint, Silvia Ienciu, Daliborca Vlad, Roxana Popescu

**Affiliations:** 1 Dept. of Internal Medicine II - Division of Nephrology, “Victor Babes” University of Medicine and Pharmacy Timisoara, Romania, Eftimie Murgu Sq. no. 2, 300041 Timisoara, RO; County Emergency Hospital Timisoara, RO.; 2 Centre for Molecular Research in Nephrology and Vascular Disease, Faculty of Medicine, “Victor Babes” University of Medicine and Pharmacy, Timisoara, Romania Eftimie Murgu Sq. no. 2, 300041 Timisoara, RO.; 3 Department of Microbiology XIV- Division of Microbiology-Virusology, ”Victor Babes” University of Medicine and Pharmacy, Timisoara, Romania, Eftimie Murgu Sq. no. 2, 300041 Timisoara, RO.; 4 Dept. of Internal Medicine II - Division of Diabetes and Metabolic Diseases, “Victor Babes” University of Medicine and Pharmacy Timisoara, Romania, Eftimie Murgu Sq. no. 2, 300041 Timisoara, RO; County Emergency Hospital Timisoara, RO.; 5 Dept. of Internal Medicine II - Division of Endocrinology, “Victor Babes” University of Medicine and Pharmacy Timisoara, Romania, Eftimie Murgu Sq. no. 2, 300041 Timisoara, RO; County Emergency Hospital Timisoara, RO.; 6 Dept. of Biochemistry and Pharmacology - Division of Pharmacology, “Victor Babes” University of Medicine and Pharmacy Timisoara, Romania, Eftimie Murgu Sq. no. 2, 300041 Timisoara, RO; County Emergency Hospital Timisoara, RO.; 7 Dept. of Functional Sciences - Division of Public Health Medicine, Faculty of Medicine, “Victor Babes” University of Medicine and Pharmacy, Timisoara, Romania, Eftimie Murgu Sq. no. 2, 300041 Timisoara, RO; County Emergency Hospital Timisoara, RO.; 8 Center for Translational Research and Systems Medicine, Faculty of Medicine, “Victor Babes” University of Medicine and Pharmacy, Timisoara, Romania, Eftimie Murgu Sq. no. 2, 300041 Timisoara, RO.; 9 Dept. of Neurosciences - Division of Neurology, “Victor Babes” University of Medicine and Pharmacy Timisoara, Romania; Eftimie Murgu Sq. no. 2, 300041 Timisoara, RO; County Emergency Hospital Timisoara, RO.; 10 Centre for Cognitive Research in Neuropsychiatric Pathology (Neuropsy-Cog), Faculty of Medicine, “Victor Babes” University of Medicine and Pharmacy, Timisoara, Romania, Eftimie Murgu Sq. no. 2, 300041 Timisoara, RO.; 11 Dept. of Anatomy and Embryology- Division of Anatomy and Embryology; “Victor Babes” University of Medicine and Pharmacy Timisoara, Romania; Eftimie Murgu Sq. no. 2, 300041 Timisoara, RO.; 12 Dept. of Surgery I- Division of Surgical Semiology I, “Victor Babes” University of Medicine and Pharmacy Timisoara, Romania; Eftimie Murgu Sq. no. 2, 300041 Timisoara, RO; Emergency Clinical Municipal Hospital Timisoara, RO.; 13 Dept. of Surgery II- Division of Surgery I, “Victor Babes” University of Medicine and Pharmacy Timisoara, Romania; Eftimie Murgu Sq. no. 2, 300041 Timisoara, RO; County Emergency Hospital Timisoara, RO.; 14 Dept. of Morphologic Microscopy - Division of Cellular and Molecular Biology; "Victor Babeș" University of Medicine and Pharmacy Timișoara, Romania; Eftimie Murgu Sq. no. 2, 300041 Timișoara, RO; County Emergency Hospital Timisoara, RO.

**Keywords:** lncRNA, miRNA, podocyte, proximal tubule, diabetes mellitus

## Abstract

**Aims:** Long noncoding RNAs (lncRNAs) play key roles in the pathophysiology of DKD involving actions of microRNAs (miRNAs). The aims of the study were to establish the involvement of selected lncRNAs in the epigenetic mechanisms of podocyte damage and tubular injury in DKD of type 2 diabetes mellitus (DM) patients in relation to a particular miRNAs profile.

**Methods:** A total of 136 patients with type 2 DM and 25 healthy subjects were assessed in a cross-sectional study concerning urinary albumin: creatinine ratio (UACR), eGFR, biomarkers of podocyte damage (synaptopodin, podocalyxin) and of proximal tubule (PT) dysfunction (Kidney injury molecule-1-KIM-1, N-acetyl-D-glucosaminidase-NAG), urinary lncRNA metastasis-associated lung adenocarcinoma transcript 1 (MALAT1), nuclear-enriched abundant transcript 1 (NEAT1), myocardial infarction-associated transcript (MIAT), taurine-upregulated gene 1 (TUG1), urinary miRNA21, 124, 93, 29a.

**Results:** Multivariable regression analysis showed that urinary lncMALAT1 correlated directly with urinary synaptopodin, podocalyxin, KIM-1, NAG, miRNA21, 124, UACR, and negatively with eGFR, miRNA93, 29a (p<0.0001; R^2^=0.727); urinary lncNEAT1 correlated directly with synaptopodin, KIM-1, NAG, miRNA21, 124, and negatively with eGFR, miRNA93, 29a (p<0.0001; R^2^=0.702); urinary lncMIAT correlated directly with miRNA93 and 29a, eGFR (p<0.0001; R^2^=0.671) and negatively with synaptopodin, KIM-1, NAG, UACR, miRNA21, 124 (p<0.0001; R^2^=0.654); urinary lncTUG1 correlated directly with eGFR, miRNA93, 29a, and negatively with synaptopodin, podocalyxin, NAG, miRNA21, 124 (p<0.0001; R^2^=0.748).

**Conclusions:** In patients with type 2 DM lncRNAs exert either deleterious or protective functions within glomeruli and PT. LncRNAs may contribute to DKD through modulating miRNAs expression and activities. This observation holds true independently of albuminuria and DKD stage.

## Introduction

Diabetic kidney disease (DKD), as a major microvascular complication of both type 1 and type 2 diabetes mellitus (DM), accounts for over 40% of patients which reach end-stage renal disease and are referred to renal replacement therapies 1.

The tubulocentric concept with regard to DKD has emphasized the pivotal role of the proximal tubule (PT) and of the tubulointerstitial compartment in the development of DKD 2. The glomerular theory raises a similar interest, with a special focus on the contribution of the podocyte injury in the course of DKD.

Particular molecular signatures and epigenetic profiles have emerged to support the complexity of DKD, pointing to the pathogenic importance of microRNAs (miRNAs) and of long noncoding RNAs (lncRNAs) in the development of DKD.

MiRNAs are endogenous small noncoding RNAs of about 20-25 nucleotides that silence the expression of target genes either through translational repression and/or mRNA degradation 3. MiRNAs play important roles in modulating gene expression and promoting the initiation and progression of various diseases, including DKD 4, 5.

MiRNAs with negative or protective roles, either at renal level or both at renal and vascular level, have been thoroughly studied in the course of DKD 4, 5, 6, 7, 8.

MiRNAs 21 and 124 have been proved to enhance podocyte injury and PT lesions 6, 7, 8, 9, 10, while other miRNAs proved to be protective at the same levels, such as miRNA 192, 125a, 126, 146a 6, 7, 8.

New key miRNAs have been proposed as protective and prompted further research, such as miRNA 93 and miRNA 29. Downregulation of miRNA 93 produces important podocyte and endothelial injury 11. A significant decrease of the miRNA 29 family expression leads to tubular lesions and severe renal fibrosis 12.

LncRNAs are a group of transcription materials with over 200 nucleotides, which lack a complete open reading frame and have no protein-coding functions 13. LncRNAs regulate gene expression, including epigenetic transcription and posttranscriptional modifications 14.

Dysregulated lncRNAs have been involved in key biological processes by mediating podocyte damage 15 and high glucose-induced PT injury 16.

Recently, a particular interest has been shown towards the intricate connection between miRNAs and lncRNAs. Cytoplasmic lncRNAs may act as molecular sponge and bind to miRNAs, thus indirectly enhancing or decreasing protein expression 17, through inhibiting their mRNA targets 18.

Recent studies in DKD revealed interactions of podocytes and PTs with lncRNAs posesing deleterious roles, among which metastasis - associated lung adenocarcinoma transcript 1 (MALAT1) and nuclear enriched abundant transcript 1 (NEAT1), and with renal protective lncRNAs, such as taurine - upregulated gene 1 (TUG1) and myocardial infarction - associated transcript (MIAT) 14.

The aim of the study was to establish the involvement of selected lncRNAs in the epigenetic mechanisms of podocyte damage and tubular injury in DKD of type 2 DM patients. The molecular mechanisms of lncRNAs intervention were evaluated in relation to a particular miRNAs profile.

## Materials and Methods

### Subjects

A total of consecutive 180 patients with type 2 DM attending the Outpatient Department of Diabetes and Metabolic Diseases (from January 2019 through December 2019), aged between 20 and 69 years, were screened for the study according to person visits and chart reviews. The inclusion criteria were DM duration higher than 5 years, and therapy with oral antidiabetic drugs (metformin, gliclazide), angiotensin-converting enzyme inhibitors or angiotensin receptor blockers, and statins. The exclusion criterion was decompensated DM (HbA_1c_ >10%). A total of 136 patients [48 patients with normoalbuminuria (group 1), 45 patients with moderately increased albuminuria (group 2), and 43 patients with severely increased albuminuria (group 3)] and 25 age- and gender-matched healthy control subjects attending a general practitioner's office for routine check-up, without known history of renal diseases and in whom diabetes or pre-diabetes were excluded by a value of HbA_1c_ ≤5.6% (group 4) were enrolled in this case series study. The remaining 48 patients out of the 73 subjects screened were excluded due to HbA_1c_ levels.

The County Emergency Hospital Ethics Committee (Board of Human Studies) approved the protocol (approval number 8/10.01.2019), and every patient provided written informed consent before enrolment.

### Laboratory assessments

The serum and urinary specimens of patients and controls utilized for the assessment of UACR, cystatin C, KIM-1, NAG, synaptopodin, and podocalyxin were frozen at -80 °C and thawed before assay. Urinary biomarkers were evaluated in the same first morning urine sample 8, 19, 20. The biomarkers of podocyte injury (synaptopodin, podocalyxin) and of PT dysfunction (KIM-1, NAG), were reported as per urinary creatinine ratio. Urinary tract infections were ruled out by negative urine cultures in all patients. CKD was defined according to the KDIGO Guideline for the Evaluation and Management of Chronic Kidney Disease 21.

### miRNA and lncRNA assessment

Total RNA was isolated from the blood and urine samples using the *mir*Vana™ miRNA Isolation Kit (Life Technologies, 5791 Van Allen Way, Carlsbad, California 92008., Cat. no. AM1560) and *microRNA Purification Kit* (Norgen Biotek Corporation, 3430 Schmon Parkway, Thorold, ON, Canada Cat. no. 29000). The quantity and quality of total RNA was verified using NanoDrop ND-1000 spectrophotometer (Analytik Jena). Total RNA was stored at -80 ºC, and reverse transcription was performed using the TaqMan MicroRNA Reverse Transcription Kit (Applied Biosystems, Foster City, CA). cDNA were amplified using the TaqMan® Universal PCR Master Mix and specific primers (TaqMan Assay) for *miRNAs (miRNA21, miRNA124, miRNA93, miRNA29a)* and lncRNAs (*lncRNA MALAT1, lncRNA NEAT1, lncRNA MIAT, lncRNA TUG1*) were utilized. The PCR reaction was performed on a 7900HT real-time PCR System. All samples were run in triplicate, and relative expression level of specific gene was calculated using the SDS software v.2.4., and U6 small nuclear RNA and GAPDH were utilized as the endogenous controls. The relative expression rate was measured by the 2^-ΔΔCT^ method (ΔΔCT=ΔCT patients-ΔCT healthy controls) 22.

### Statistical analysis

Clinical and biological data are presented as medians and interquartile range (IQR), as for variables with skewed distribution. Depending on the distribution of the values, differences between subgroups were analyzed with the Mann-Whitney U test for comparison of 2 groups and the Kruskal-Wallis test for comparison of 4 groups. Univariable and multivariable linear regression analyses were conducted in order to assess the significance of the relation of serum and urinary lncRNAs with serum and urinary miRNAs, as well as with other continuous variables. The statistical methods were utilised as required by a case series study which is an exploratory study. Case series have a descriptive study design. In our case series analyses were performed in order to investigate the association between lncRNAs and a particular miRNA profile. Statistical significance was set at p<0.05 and the analyses applied were conducted with Stata 15.1 (Statacorp, Texas, USA).

## Results

Demographic, clinical, and biological data of the study subjects are presented in Table 1 as medians and IQR. In Table 1, p values were corrected for multiple comparisons.

### LncRNA MALAT1 and lncRNA NEAT1 are upregulated in early DKD and inhibit miRNA 93 and miRNA 29a function, while enhancing miRNA 21 and miRNA 124 activities

In univariable linear regression analysis, we found a direct relationship between urinary and serum lncRNA MALAT1, and urinary and serum miRNA 21, miRNA 124, urinary podocalyxin, urinary synaptopodin, urinary KIM-1, urinary NAG, and UACR (p<0.001). This lncRNA showed negative correlations with urinary and serum miRNA 93 and miRNA 29a, as well as with eGFR (p<0.001). Urinary and serum lncRNA NEAT1 followed the same trend as lncRNA MALAT1 (Table 2 and 3).

Multivariable regression analysis yielded models which showed that urinary lncRNA MALAT1 correlated indirectly with urinary miRNA 29a and eGFR, and directly with UACR (R^2^ = 0.80). Also, we found a direct relationship between urinary lncRNA NEAT1 and urinary miRNA 21, urinary podocalyxin, KIM-1, and UACR, and an indirect relationship with urinary miRNA 93 (R^2^ = 0.74) (Table 4).

Serum lncRNA MALAT1 showed direct correlations with serum miRNA 124 and indirect correlations with serum miRNA 29a and eGFR (R^2^ = 0.94). Serum lncRNA NEAT1 correlated directly with urinary podocalyxin, KIM-1, and UACR, and indirectly with serum miRNA 29a and eGFR (R^2^ = 0.76) (Table 5).

These data point to the deleterious involvement of lncRNA MALAT1 and lncRNA NEAT1 upon the glomeruli and the PT in early DKD, as documented by the direct correlation of these lncRNAs with podocyte damage and PT dysfunction biomarkers. Their negative effects are also supported by the indirect correlations with eGFR and the two miRNAs, miRNA 93 and miRNA 29a, respectively, known for their protective effects in the course of DKD.

### LncRNA MIAT and lncRNA TUG1 are downregulated and enhance miRNA 93 and miRNA 29a expression in DKD patients, and suppress miRNA 21 and miRNA 124 activities

In univariable linear regression analysis, we found a direct relationship between urinary and serum lncRNA MIAT, and urinary and serum miRNA 93 and miRNA 29a, as well as eGFR, and an indirect relationship with urinary and serum miRNA 21, miRNA 124, UACR, urinary podocalyxin, urinary synaptopodin, KIM-1, and NAG. Urinary and serum lncRNA-TUG1 displayed similar correlations with lncRNA MIAT (Table 2 and 3).

Multivariable regression analysis produced models in which urinary lncRNA MIAT correlated directly with urinary miRNA 93 and urinary miRNA 29a, and indirectly with UACR (R^2^= 0.66). Urinary lncRNA TUG1 correlated positively with urinary miRNA 93, miRNA 29a and eGFR, and indirectly with urinary miRNA 124 and KIM-1 (R^2^= 0.84) (Table 4).

Serum lncRNA MIAT displayed direct correlations with serum miRNA 93, miRNA 29a, and eGFR, and indirect correlations with serum miRNA 21 (R^2^= 0.75). Serum lncRNA TUG1 correlated positively with serum miRNA29a and eGFR, and negatively with serum miRNA 21 (R^2^= 0.73) (Table 5).

The results presented show that lncRNA MIAT and lncRNA TUG1 enhance the expression and potentiate the protective effects of miRNA 93 and miRNA 29a. Moreover, these lncRNAs display protective activities upon the podocytes and the PT, as supported by their indirect correlations with the podocyte injury and PT dysfunction biomarkers.

## Discussion

Interactions among multiple molecules and signaling pathways contribute to the pathogenesis and progression of DKD. LncRNAs play key roles in the pathophysiology of DKD, involving actions of miRNAs. The present study aimed at establishing the involvement of selected lncRNAs in the epigenetic mechanisms of DKD.

We found that in patients with type 2 DM lncRNAs exert distinct functions in different cell types, either deleterious or protective, at both glomerular and tubular level. These properties were displayed through modulating miRNAs expression and activities.

### LncRNA MALAT1 and lncRNA NEAT1 are dysregulated in DKD and involved in podocyte and PT injury via miRNAs modulation

*LncRNA MALAT1* is highly expressed in the kidney 23 and significantly upregulated in early DKD 24. This lncRNA is an initiator of inflammation and oxidative stress through upregulation of several inflammatory mediators 14, 25. LncRNA MALAT1 could be attributed severe podocyte damage via its interplay with β-catenin, a key mediator of podocyte injury and renal fibrosis 15. LncRNA MALAT1 is upregulated in PT cells 16, 26 and intervenes in PT dysfunction by regulating tubular cell viability 27, 23, 24.

In our study, lncRNA MALAT1 was upregulated and exerted negative effects upon the podocytes and the PT, as supported by the association with the levels of podocyte damage and PT dysfunction biomarkers.

LncRNAs regulate miRNAs through two major modes of regulation, either by enhancing or by inhibiting their function 28. Profibrotic miRNAs, such as miRNA 21 9 and miRNA 124, may exert a role in podocyte damage under mechanical stress 10. In our study, serum and urinary miRNA 21 was upregulated and was associated with podocyte and PT injury biomarkers, a fact which points to its potential involvement within the glomerulus and the tubulo-interstitial compartment. These data are in keeping with other studies in which miRNA 21 has been ascribed pro-fibrotic and pro-inflammatory properties 6, 7, 8, 29. The same trend was followed by miRNA 124 which may impair podocyte structural and functional integrity, and may induce tubulointerstitial fibrosis 6, 7, 8, 10. LncRNA MALAT1 enhanced the activity of both miRNA 21 and miRNA 124 across all the groups of patients with type 2 DM studied, even in the early stage of DKD.

MiRNA 93 is downregulated in the podocytes and tubular cells of DKD patients 11, 30 and plays a key role in preserving podocyte integrity 30 and in preventing renal interstitial fibrosis in DKD 31. Although miRNA 29 family displays intriguing roles in DKD, its expression being both increased and decreased 32, miRNA 29a is considered a renoprotective miRNA involved in inflammation-mediated podocyte and tubular injury under diabetic conditions 12, 33, 34, 35.

In our patients, lncRNA MALAT1 decreased the expression of miRNA 93 and miRNA 29a. This observation was concurrent with the increased levels of podocyte injury and of PT dysfunction biomarkers, even in normoalbuminuric patients.

*LncRNA NEAT1* is known to intervene in fibrogenesis by extracellular matrix accumulation and epithelial-mesenchymal transition in DKD 36, 37. In our study, lncRNA NEAT1 increased the expression of both miRNA 21 and miRNA 124, and decreased the activity of miRNA 93 and miRNA 29a, irrespective of UACR and level of renal function. This data is in keeping with the results derived from experimental 36 and clinical studies 37, which show that these effects of lncRNA NEAT1 are exerted through mediating miRNAs expression and activities.

The deleterious properties of lncRNA NEAT1 were extended at both glomerular and tubular level, as supported by its association with podocyte damage and PT dysfunction biomarkers. A recent study by Li N et al demonstrated that lncRNA NEAT1 facilitated proliferation, fibrosis, and epithelial-mesenchymal transition through sponging miRNA 23c in diabetic nephropathy. In their study the authors approached the PT dysfunction biomarkers, KIM-1 and NGAL, by assessing the expression of their mRNA in serum, which proved to be enhanced in parallel with lncRNA NEAT1 activities 38.

### LncRNA MIAT and lncRNA TUG1 exert renoprotective functions in DKD through interactions with specific miRNAs

Among other lncRNAs which act as regulators of diabetic tubular injury in DKD 39, 40, 41, the expression of *lncRNA MIAT* was reduced and negatively correlated with serum creatinine and blood urea nitrogen levels 24, 42. It is assumed that lncRNAs could be potential regulators of high glucose-induced renal tubular injury. In the study by Zhou L et al, lncRNA MIAT was decreased in the proximal tubules homogenate of STZ-induced diabetic kidneys from Wistar rats. LncRNA MIAT expression correlated negatively with the biomarkers of kidney function. The authors showed that lncRNA MIAT may regulate PT cell viability via stabilizing nuclear factor erythroid 2-related factor 2 expression, which is the key molecule of cellular defense against high blood glucose - induced oxidative stress 16.

In our study, lncRNA MIAT was downregulated and showed a strong association with decreased levels of podocyte injury and PT dysfunction biomarkers. We hypothesized that lncRNA MIAT could exert protective effects at both glomerular and tubular level. Furthermore, due to the statistically significant association of lncRNA MIAT with the enhanced expression of miRNA 93 and miRNA 29a, and with the decreased activity of miRNA 21 and miRNA 124, we speculated that lncRNA MIAT could modulate the activity of these miRNAs. As a direct consequence of lncRNA MIAT intervention, the protective miRNAs 93 and 29a could prevail, thus promoting the mechanism of defense against podocyte and PT injury from the early stages of DKD.

*LncRNA TUG1* plays a key role in the initiation and progression of DKD 14. LncRNA TUG1 is significantly repressed in the podocytes and produces metabolic alterations in these cells. This lncRNA regulates the peroxisome proliferator-activated receptor γ co-activator α (PGC-1α), thus rescuing this important member of the nuclear receptor superfamily 14.

LncRNA TUG1 participated in the regulation of mitochondrial function in podocytes in a murine model of DKD 14, phenomenon regulated by PGC-1α, known for its role in mitochondrial bioenergetics and respiration 43. The modulation of mitochondrial metabolism by lncRNA TUG1 was documented by Long J et al in a mouse model through recruitment of PGC-1α to its own promoter 44. By interacting with miRNAs, lncRNA TUG 1 may interfere with extracellular matrix accumulation and pro-inflammatory cytokines secretion in DKD 14.

In our study, lncRNA TUG1 paralleled the activities of lncRNA MIAT, thus pointing to its protective properties in the course of DKD, even in normoalbuminuric patients. Our results are in keeping with those provided by the experimental study by Wang F et al, in which the authors reported that lncRNA TUG1 can improve renal fibrosis in diabetic nephropathy by modulating miRNA 21 targeting tissue inhibitor of metalloproteinase 3 (TIMP 3). The intervention of lncRNA TUG1 via miRNA 21 modulation regulates TIMP3, known as a potent inhibitor of metalloproteinase 3 45.

There are several limitations of our study. First, the small sample size of the patients studied may reduce the statistical power of the study. Second, cell-specific and tissue-specific expression and activities of miRNAs and lncRNAs could interfere interpretation of data with regard to their interactions. Finally, the study follows the design of a case series study, which requires validation in a longitudinal study, in order to prove a statistically significant relation of causality between the miRNAs and the lncRNAs studied.

The strength of our study derives from the documentation in a human study of an important association of lncRNAs which poses well defined roles in the pathogenesis of DKD, such as lncRNA MALAT1, NEAT1, MIAT, and TUG1, with miRNAs which have concurrent negative (miRNA 21, 124), or positive (miRNA 93, 29a) effects, respectively, even in the early stages of DKD.

In conclusion, in patients with type 2 DM, lncRNAs exert distinct functions in different cell types, either deleterious, as was the case with lncRNA MALAT1 and lncRNA NEAT1, or protective, as driven by lncRNA MIAT and lncRNA TUG1.

These properties were documented at both glomerular and PT level due to their strong association with podocyte damage and PT dysfunction biomarkers. LncRNAs may contribute to initiation and DKD progression through modulating miRNAs expression and activities.

The results of our study forward several lncRNAs and miRNAs as candidate biomarkers in the early diagnosis of DKD. The study is based on translational research from epigenetic mechanisms to clinical practice, which may enable a precision medicine approach to therapeutic strategies in the early stages of renal involvement in the course of DM. The interactions between these molecular pathways could provide a theoretical basis which may allow for a tailored therapy of DKD in its early stages.

## Figures and Tables

**Table 1:** Clinical and biological data of the patients studied

Parameter	Group 1 (healthy controls)	Group 2 (normoalbuminuria)	Group 3 (moderately increased albuminuria)	Group 4 (severely increased albuminuria)
Number of subjects	25	48	45	43
Age (years)	61 (55, 69)	59 (53.5;63)	60 (55;67)	60 (57;66)
DM duration (years)		8 (7;10) ^♣^	8 (6;13)	10 (8;13)
BMI	26.67 (23.88;29.76)^ #^	32.02 (29.05;35.06) ^⁋^	32 (28;38.09)	34 (30;37)
SBP (mmHg)	120 (120;130)^ #^	130 (125;140)^ †^	130 (125;140) ^♦^	150 (145;170)
DBP (mmHg)	70 (70;80)^ #^	75 (70;80)^ †^	75 (70;80)^ ♦^	85 (75;90)
Hb (g/dl)	13.6 (12.5;14.5)^ #^	12.65 (12;13.65)^ †^	13.4 (12.2;14)^ ♦^	11.25 (10.5;12.9)
Serum creatinine (mg/dl)	0.92 (0.78;1.01)^ #^	0.78 (0.69;0.87)^ †^	0.74 (0.69;0.81)^ ♦^	1.65 (1.47;1.94)
eGFR (ml/min/1.73m^2^)	99.35 (91.46; 105.27)^ #^	39.60 (36.44;99.77)	48.89 (46.30;52.70)^ ♦^	43.40 (36.49;46.55)
Glycaemia (fasting) (mg/dl)	99 (90;100)^ #^	150.5 (117;203) ^⸿, ‡^	165 (125;264)	176 (140;238)
HbA_1c_ (%)	5.8 (5.7;6)^ #^	6.95 (6.5;7.4)^ *, †^	7.8 (6.94;9.51)	8.43 (7.69;9.67)
Cholesterol (mg/dl)	160 (125; 181)^ #^	203 (180;243)^ ∆^	202 (172;288)	223 (187;276)
Triglycerides (mg/dl)	132 (107;148)^ ¤^	161 (129;191.5)	158 (117;205)	167 (122;236)
hsCRP (mg/dl)	0.85 (0.8;2.78)^ #^	3.25 (2.31;4.75)^ ●, ∏^	10 (8.55;16.87)	18.81 (2.6;27.68)
UACR (mg/g)	10.82 (10.12;14.32)^ #^	18.38 (16.63;20.01)^ ●, †^	83.33 (44.28;117.38)^ ♦^	541.45 (486.34;981.91)
Cystatin C (mg/L)	0.66 (0.57;0.67)^ #^	3 (0.72;3.24) ^⁑, ◊^	2.1 (1.94;2.23)^ ♦^	1.46 (1.31;1.53)
Nephrin/creat (mg/g)	0.08 (0.04;0.09)^ #^	0.12 (0.11;0.15)^ ●, †^	0.78 (0.53;1.1)^ ♦^	6.25 (4.6;7.2)
Podocalyxin/creat (mg/g)	32.5 (28.6;38.4)^ #^	65.6 (58.9;70.35)^ ●, †^	121.6 (114.2;155.7)^ ♦^	870.3 (384.9;950.1)
Synaptopodin/creat (mg/g)	9.33 (6.88;9.64)^ #^	18.6 (15.38;21.34)^ ●, †^	26.58 (24.89;27.65)^ ♦^	130.5 (72.4;137.78)
KIM-1/creat (ng/g)	43.82 (26;47.5)^ #^	74.18 (63.6;87.28)^ ●, †^	120.56 (114.73;133.78)^ ♦^	684.83 (570.4;831.65)
NAG/creat (UI/g)	2.22 (1.69;2.41)^ #^	3.17 (1.88;5.28)^ ●, †^	10.5 (9.92;11.96)^ ♦^	16.74 (16.05;18)
Alpha -1 microglobulin	3.16 (2.49; 3.24)^ #^	4.16 (3.76;5.16)^ ●, †^	7.73 (6.71;9.08)^ ♦^	56.27 (31.18;65)
u-miRNA-29a	2.95 (2.75;3.08)^ #^	1.80 (1.7;2.03)^ ●, †^	0.76 (0.6;0.91)^ ♦^	0.47 (0.41;0.6)
p-miRNA-29a	3.64 (3.52;3.75)^ #^	2.64 (2.53;2.73)^ ●, †^	1.54 (1.39;1.71)^ ♦^	0.91 (0.78;0.97)
u-miRNA-21p	1.02 (0.92;1.02)^ #^	1.19 (1.14;1.26)^ ●, †^	1.82 (1.67;1.90)^ ♦^	2.67 (2.27;2.75)
p-miRNA-21p	1.09 (1.06;1.17)^ #^	1.26 (1.16;1.44)^ ●, †^	2.34 (2.25;2.54)^ ♦^	2.94 (2.85;3.04)
u-miRNA-124	0.54 (0.44;0.73)^ #^	1.5 (1.25;1.71)^ ●, †^	2.74 (2.57;2.85)^ ♦^	3.23 (3.18;3.37)
p-miRNA-124	0.81 (0.77;0.96)^ #^	1.95 (1.67;2.44)^ ●, †^	3.38 (3.07;3.59)^ ♦^	3.86 (3.79;4.01)
u-miRNA-93	2.21 (1.9;3.25)^ #^	2.89 (2.20;3.08)^ ●, †^	1.7 (1.38;1.92)^ ♦^	1.14 (0.89;1.25)
p-miRNA-93	3.45 (3.24;4.49)^ #^	2.98 (2.4;3.93)^ ●, †^	2.42 (2.26;2.65)^ ♦^	1.44 (1.21;1.57)
u-lnc-MALAT-1	1.13 (1.1;1.28)^ #^	2.3 (1.89;2.85)^ ●, †^	3.53 (3.08;3.88)^ ♦^	3.88 (3.66;4.23)
p-lnc-MALAT-1	1.61 (1.54;1.64)^ #^	2.51 (2.13;2.83)^ ●, †^	4.04 (3.64;4.46)^ ♦^	4.53 (4.39;4.72)
u-lnc-NEAT	0.78 (0.68;0.82)^ #^	0.90 (0.68;1.03)^ ●, †^	1.24 (1.10;1.57)^ ♦^	2.27 (1.92;2.53)
p-lnc-NEAT	1.06 (1;1.13)^ #^	1.38 (1.19;1.54)^ ●, †^	1.67 (1.53;2.01)^ ♦^	2.82 (2.19;3.1)
u-lnc-MIAT	2.53 (2.4;2.91)^ #^	1.77 (1.37;2.07)^ ▀, †^	1.4 (1.26;1.84)^ ♦^	1.03 (0.62;1.16)
p-lnc-MIAT	2.77 (2.61;3.12)^ #^	2.67 (1.73;2.86)^ ●, †^	1.83 (1.43;1.95)^ ♦^	1.28 (0.95;1.38)
u-lnc-TUG	3.13 (2.84;3.21)^ #^	2.65 (2.18;3.31)^ ●, †^	1.99 (1.7;2.24)^ ♦^	1.32 (1.05;1.41)
p-lnc-TUG	3.38 (3.09;3.57)^ #^	3.10 (2.67;3.77)^ ●, †^	2.46 (2.14;2.62)^ ♦^	2.07 (1.75;2.26)

Clinical and biological data are presented as medians and IQR, as for variables with skewed distribution. #: Significance between group 1 vs group 2 vs group 3 vs group 4; p < 0.001; ¤: Significance between group 1 vs group 2 vs group 3 vs group 4; p = 0.001; ● Significance between group 2 vs group 3; p < 0.001; ⸿: Significance between group 2 vs group 3; p = 0.044; *Significance between group 2 vs group 3; p = 0.001; ⁑ Significance between group 2 vs group 3; p = 0.015; ▀ Significance between group 2 vs group 3; p = 0.030; † Significance between group 2 vs group 4; p < 0.001; ♣ Significance between group 2 vs group 4; p = 0.009; ⁋ Significance between group 2 vs group 4; p = 0.047; ‡ Significance between group 2 vs group 4; p = 0.014; ∆: Significance between group 2 vs group 4; p = 0.046; ^∏^ Significance between group 2 vs group 4; p = 0.006; ◊ Significance between group 2 vs group 4; p = 0.016; ♦ Significance between group 3 vs group 4; p < 0.001. BMI: body mass index; SBP: systolic blood pressure; DBP: diastolic blood pressure; DM: diabetes mellitus; eGFR: estimated glomerular filtration rate; hsCRP: high-sensitive C reactive protein; UACR: urinary albumin: creatinine ratio; KIM-1/creat: urinary kidney injury molecule-1:creatinine ratio; NAG/creat: urinary *N*-beta-D-acetyl-glucosaminidase/creat; Nephrin/creat: Nephrin/creatinine ratio; Podocalyxin/creat: Podocalyxin/creatinine ratio; Synaptopodin/creat: Synaptopodin/creatinine ratio; Hb: serum haemoglobin; HbA1C: glycated haemoglobin; p-miRNA: plasmatic microRNA; u-miRNA: urinary microRNA; u-lncRNA: urinary long noncoding RNA; p-lncRNA: plasmatic long noncoding RNA; MALAT-1: metastasis associated lung adenocarcinoma transcript 1 gene; MIAT: myocardial infarction associated transcript gene; NEAT: nuclear enrich abundant transcript gene; TUG: taurine upregulated gene.

**Table 2:** Univariable regression analysis for urinary lnc-RNA

Parameter	Variable	R^2^	Coef β	P
u-lnc- MALAT-1	u-miRNA-29a	0.78	-1.003	<0.001
	p-miRNA-29a	0.78	-0.911	<0.001
	u-miRNA-21p	0.62	1.295	<0.001
	p-miRNA-21p	0.64	1.063	<0.001
	u-miRNA-124	0.73	0.885	<0.001
	p-miRNA-124	0.81	0.825	<0.001
	u-miRNA-93	0.34	-0.678	<0.001
	p-miRNA-93	0.44	-0.658	<0.001
	Podocalyxin/creat	0.35	0.001	<0.001
	Synaptopodin/creat	0.36	0.013	<0.001
	KIM-1/creat	0.35	0.002	<0.001
	NAG/creat	0.46	0.102	<0.001
	UACR	0.34	0.001	<0.001
	eGFR	0.25	-0.018	<0.001
u-lnc-NEAT	u-miRNA-29a	0.47	-0.473	<0.001
	p-miRNA-29a	0.59	-0.476	<0.001
	u-miRNA-21p	0.62	0.784	<0.001
	p-miRNA-21p	0.57	0.602	<0.001
	u-miRNA-124	0.54	0.458	<0.001
	p-miRNA-124	0.52	0.400	<0.001
	u-miRNA-93	0.42	-0.450	<0.001
	p-miRNA-93	0.40	-0.379	<0.001
	Podocalyxin/creat	0.62	0.001	<0.001
	Synaptopodin/creat	0.59	0.010	<0.001
	KIM-1/creat	0.61	0.001	<0.001
	NAG/creat	0.40	0.058	<0.001
	UACR	0.42	0.001	<0.001
	eGFR	0.21	-0.010	<0.001
u-lnc-MIAT	u-miRNA-29a	0.58	0.618	<0.001
	p-miRNA-29a	0.59	0.562	<0.001
	u-miRNA-21p	0.47	-0.806	<0.001
	p-miRNA-21p	0.47	-0.644	<0.001
	u-miRNA-124	0.55	-0.549	<0.001
	p-miRNA-124	0.52	-0.470	<0.001
	u-miRNA-93	0.19	0.361	<0.001
	p-miRNA-93	0.30	0.388	<0.001
	Podocalyxin/creat	0.27	-0.001	<0.001
	Synaptopodin/creat	0.33	-0.009	<0.001
	KIM-1/creat	0.30	-0.001	<0.001
	NAG/creat	0.33	-0.062	<0.001
	UACR	0.38	-0.001	<0.001
	eGFR	0.17	0.011	<0.001
u-lnc-TUG	u-miRNA-29a	0.64	0.703	<0.001
	p-miRNA-29a	0.70	0.671	<0.001
	u-miRNA-21p	0.65	-1.030	<0.001
	p-miRNA-21p	0.72	-0.870	<0.001
	u-miRNA-124	0.70	-0.671	<0.001
	p-miRNA-124	0.57	-0.539	<0.001
	u-miRNA-93	0.55	0.659	<0.001
	p-miRNA-93	0.43	0.508	<0.001
	Podocalyxin/creat	0.45	-0.001	<0.001
	Synaptopodin/creat	0.48	-0.011	<0.001
	KIM-1/creat	0.47	-0.001	<0.001
	NAG/creat	0.51	-0.083	<0.001
	UACR	0.48	-0.001	<0.001
	eGFR	0.11	0.009	<0.001

UACR: urinary albumin:creatinine ratio; eGFR: estimated glomerular filtration rate; KIM-1/creat: urinary kidney injury molecule-1:creatinine ratio; NAG/creat: urinary *N*-beta-D-acetyl-glucosaminidase/creat; Podocalyxin/creat: Podocalyxin/creatinine ratio; Synaptopodin/creat: Synaptopodin/creatinine ratio; p-miRNA: plasmatic microRNA; u-miRNA: urinary microRNA; u-lncRNA: urinary long noncoding RNA; MALAT-1: metastasis associated lung adenocarcinoma transcript 1 gene; MIAT: myocardial infarction associated transcript gene; NEAT: nuclear enrich abundant transcript gene; TUG: taurine upregulated gene.

**Table 3:** Univariable regression analysis for serum lnc-RNA

Parameter	Variable	R^2^	Coef β	P
p-lnc-MALAT-1	u-miRNA-29a	0.86	-1.191	<0.001
	p-miRNA-29a	0.88	-1.094	<0.001
	u-miRNA-21p	0.71	1.568	<0.001
	p-miRNA-21p	0.76	1.300	<0.001
	u-miRNA-124	0.83	1.067	<0.001
	p-miRNA-124	0.91	0.990	<0.001
	u-miRNA-93	0.47	-0.895	<0.001
	p-miRNA-93	0.48	-0.776	<0.001
	Podocalyxin/creat	0.40	0.002	<0.001
	Synaptopodin/creat	0.43	0.016	<0.001
	KIM-1/creat	0.40	0.002	<0.001
	NAG/creat	0.55	0.126	<0.001
	UACR	0.40	0.002	<0.001
	eGFR	0.35	-0.025	<0.001
p-lnc-NEAT	u-miRNA-29a	0.51	-0.527	<0.001
	p-miRNA-29a	0.62	-0.528	<0.001
	u-miRNA-21p	0.63	0.852	<0.001
	p-miRNA-21p	0.56	0.648	<0.001
	u-miRNA-124	0.57	0.509	<0.001
	p-miRNA-124	0.56	0.447	<0.001
	u-miRNA-93	0.40	-0.477	<0.001
	p-miRNA-93	0.41	-0.416	<0.001
	Podocalyxin/creat	0.63	0.001	<0.001
	Synaptopodin/creat	0.62	0.011	<0.001
	KIM-1/creat	0.60	0.001	<0.001
	NAG/creat	0.42	0.063	<0.001
	UACR	0.41	0.001	<0.001
	eGFR	0.29	-0.013	<0.001
p-lnc-MIAT	u-miRNA-29a	0.64	0.716	<0.001
	p-miRNA-29a	0.65	0.653	<0.001
	u-miRNA-21p	0.55	-0.959	<0.001
	p-miRNA-21p	0.54	-0.764	<0.001
	u-miRNA-124	0.60	-0.630	<0.001
	p-miRNA-124	0.51	-0.516	<0.001
	u-miRNA-93	0.26	0.458	<0.001
	p-miRNA-93	0.35	0.464	<0.001
	Podocalyxin/creat	0.31	-0.001	<0.001
	Synaptopodin/creat	0.35	-0.010	<0.001
	KIM-1/creat	0.32	-0.001	<0.001
	NAG/creat	0.42	-0.077	<0.001
	UACR	0.35	-0.001	<0.001
	eGFR	0.09	0.009	<0.001
p-lnc-TUG	u-miRNA-29a	0.55	0.567	<0.001
	p-miRNA-29a	0.58	0.530	<0.001
	u-miRNA-21p	0.53	-0.804	<0.001
	p-miRNA-21p	0.63	-0.706	<0.001
	u-miRNA-124	0.59	-0.536	<0.001
	p-miRNA-124	0.46	-0.420	<0.001
	u-miRNA-93	0.48	0.537	<0.001
	p-miRNA-93	0.34	0.390	<0.001
	Podocalyxin/creat	0.31	-0.001	<0.001
	Synaptopodin/creat	0.32	-0.008	<0.001
	KIM-1/creat	0.31	-0.001	<0.001
	NAG/creat	0.42	-0.065	<0.001
	UACR	0.35	-0.001	<0.001
	eGFR	0.05	0.006	<0.001

UACR: urinary albumin:creatinine ratio; eGFR: estimated glomerular filtration rate; KIM-1/creat: urinary kidney injury molecule-1:creatinine ratio; NAG/creat: urinary *N*-beta-D-acetyl-glucosaminidase/creat; Podocalyxin/creat: Podocalyxin/creatinine ratio; Synaptopodin/creat: Synaptopodin/creatinine ratio; p-miRNA: plasmatic microRNA; u-miRNA: urinary microRNA; p-lncRNA: plasmatic long noncoding RNA; MALAT-1: metastasis associated lung adenocarcinoma transcript 1 gene; MIAT: myocardial infarction associated transcript gene; NEAT: nuclear enrich abundant transcript gene; TUG: taurine upregulated gene.

**Table 4:** Multivariable regression analysis for urinary lnc-RNA

Parameter	Variable	Coef β	P	95% CI	Prob>F	R^2^
u-lnc-MALAT-1	Constant	3.963	0.0001	3.750 to 4.175	0.00001	0.80
	u-miRNA-29a	-1.027	0.0001	-1.149 to -0.906		
	UACR	0.0003	0.011	0.00007 to 0.0005		
	eGFR	0.004	0.007	0.001 to 0.008		
u-lnc-NEAT	Constant	0.670	0.0001	0.327 to 1.013	0.00001	0.74
	u-miRNA-21p	0.352	0.0001	0.189 to 0.516		
	u-miRNA-93	-0.103	0.011	-0.182 to -0.024		
	Podocalyxin/creat	0.0006	0.0001	0.0003 to 0.0009		
	KIM-1/creat	0.0006	0.0001	0.0002 to 0.001		
	UACR	-0.0003	0.003	-0.0006 to -0.0001		
u-lnc-MIAT	Constant	1.422	0.0001	1.191 to 1.653	0.00001	0.66
	u-miRNA-29a	0.599	0.0001	0.496 to 0.702		
	u-miRNA-93	-0.217	0.0001	-0.324 to -0.111		
	UACR	-0.0007	0.0001	-0.0009 to -0.0004		
u-lnc-TUG	Constant	2.528	0.0001	1.828 to 3.229	0.00001	0.84
	u-miRNA-29a	0.364	0.0001	0.184 to 0.544		
	u-miRNA-124	-0.274	0.005	-0.465 to -0.083		
	u-miRNA-93	0.272	0.0001	0.186 to 0.358		
	KIM-1/creat	-0.0005	0.0001	-0.0007 to -0.0003		
	eGFR	-0.011	0.0001	-0.013 to -0.008		

UACR- urinary albumin:creatinine ratio; eGFR-estimated glomerular filtration rate; KIM-1/creat-urinary kidney injury molecule-1:creatinine ratio; Podocalyxin/creat - Podocalyxin/creatinine ratio; u-miRNA- urinary microRNA; u-lncRNA - urinary long noncoding RNA; MALAT-1 - metastasis associated lung adenocarcinoma transcript 1 gene; MIAT - myocardial infarction associated transcript gene; NEAT - nuclear enrich abundant transcript gene; TUG - taurine upregulated gene.

**Table 5:** Multivariable regression analysis for serum lnc-RNA

Parameter	Variable	Coef β	P	95% CI	Prob>F	R^2^
p-lnc-MALAT-1	Constant	1.183	0.002	0.447 to 1.919	0.00001	0.94
	p-miRNA-29a	-0.294	0.0001	-0.445 to -0.144		
	p-miRNA-124	0.864	0.0001	0.719 to 1.009		
	eGFR	0.007	0.0001	0.004 to 0.009		
p-lnc NEAT	Constant	2.102	0.0001	1.888 to 2.316	0.00001	0.76
	p-miRNA-29a	-0.237	0.0001	-0.332 to -0.142		
	Podocalyxin/creat	0.0008	0.0001	0.0005 to 0.001		
	KIM-1	0.0006	0.001	0.0002 to 0.0009		
	UACR	-0.0002	0.034	-0.0005 to -0.00002		
	eGFR	-0.002	0.048	-0.005 to -0.00002		
p-lnc-MIAT	Constant	-0.089	0.815	-0.842 to -0.663	0.00001	0.75
	p-miRNA-29a	0.995	0.0001	0.812 to 1.177		
	p-miRNA-21p	0.264	0.015	0.052 to 0.476		
	p-miRNA-93	0.108	0.021	0.016 to 0.200		
	eGFR	-0.012	0.0001	-0.016 to -0.009		
p-lnc-TUG	Constant	3.026	0.0001	2.390 to 3.661	0.00001	0.73
	p-miRNA-29a	0.461	0.0001	0.299 to 0.624		
	p-miRNA-21p	-0.352	0.0001	-0.536 to -0.167		
	eGFR	-0.010	0.0001	-0.012 to -0.007		

UACR- urinary albumin:creatinine ratio; eGFR-estimated glomerular filtration rate; KIM-1/creat-urinary kidney injury molecule-1:creatinine ratio; Podocalyxin/creat - Podocalyxin/creatinine ratio; p-miRNA- plasmatic microRNA; p-lncRNA - plasmatic long noncoding RNA; MALAT-1 - metastasis associated lung adenocarcinoma transcript 1 gene; MIAT - myocardial infarction associated transcript gene; NEAT - nuclear enrich abundant transcript gene; TUG - taurine upregulated gene.

## References

[1] United States Renal Data System. 2019 USRDS annual data report: Epidemiology of kidney disease in the United States. National Institutes of Health, National Institute of Diabetes and Digestive and Kidney Diseases, Bethesda, MD, 2019.

[2] Vallon V, Thomson SC (2012). Renal function in diabetic disease models: the tubular system in the pathophysiology of the diabetic kidney. Annu Rev Physiol.

[3] Kato M (2018). Noncoding RNAs as therapeutic targets in early-stage diabetic kidney disease. Kidney Res Clin Pract.

[4] Bhatt K, Lanting L, Jia Y, Yadav S, Reddy M, Magilnick N (2016). Anti-inflammatory role of microRNA-146a in the pathogenesis of diabetic nephropathy. J Am Soc Nephrol.

[5] Trionfini P, Benigni A (2017). MicroRNAs as master regulators of glomerular function in health and disease. J Am Soc Nephrol.

[6] Petrica L, Pusztai A-M, Vlad M, Vlad A, Gadalean F, Dumitrascu V (2020). MiRNA Expression is Associated with Clinical Variables Related to Vascular Remodeling in the Kidney and the Brain in Type 2 Diabetes Mellitus Patients. Endocr Res.

[7] Petrica L, Milas O, Vlad M, Vlad A, Gadalean F, Dumitrascu V (2019). Interleukins and miRNAs intervene in the early stages of diabetic kidney disease in Type 2 diabetes mellitus patients. Biomark Med.

[8] Milas O, Gadalean F, Vlad A, Dumitrascu V, Gluhovschi C, Gluhovschi G (2018). Deregulated profiles of urinary micro-RNAs may explain podocyte injury and proximal tubule dysfunction in normoalbuminuric patients with type 2 diabetes mellitus. J Investig Med.

[9] Wang J, Gao Y, Ma M, Li M, Zou D, Yang J (2013). Effect of miR-21 on renal fibrosis by regulating MMP-9 and TIMP1 in kk-ay diabetic nephropathy mice. Cell Biochem Biophys.

[10] Li D, Lu Z, Jia J, Zheng Z, Lin S (2013). Changes in microRNAs associated with podocytic adhesion damage under mechanical stress. J Renin Angiotensin Aldosterone Syst.

[11] Badal SS, Wang Y, Long J, Corcoran DL, Chang BH, Truong LD (2016). MiR-93 regulates Msk2-mediated chromatin remodelling in diabetic nephropathy. Nat Commun.

[12] Wang B, Komers R, Carew R, Winbanks CE, Xu B, Herman-Edelstein M (2012). Suppression of microRNA-29 expression by TGF-β1 promotes collagen expression and renal fibrosis. J Am Soc Nephrol.

[13] Kapranov P, Cheng J, Dike S, Nix DA, Duttagupta R, Willingham AT (2007). RNA maps reveal new RNA classes and a possible function for pervasive transcription. Science.

[14] Li Y, Xu K, Xu K, Chen S, Cao Y, Zhan H (2019). Roles of identified long noncoding RNA in diabetic nephropathy. J Diabetes Res.

[15] Hu M, Wang R, Li X, Fan M, Lin J, Zhen J (2017). LncRNA MALAT1 is dysregulated in diabetic nephropathy and involved in high glucose-induced podocyte injury via its interplay with β-catenin. J Cell Mol Med.

[16] Zhou L, Xu DY, Sha WG, Shen L, Lu GY, Yin X (2015). Long non-coding MIAT mediates high glucose-induced renal tubular epithelial injury. Biochem Biophys Res Commun.

[17] Kashi K, Henderson L, Bonetti A, Carninci P (2016). Discovery and functional analysis of lncRNAs: Methodologies to investigate an uncharacterized transcriptome. Biochim Biophys Act.

[18] Paraskevopoulou MD, Hatzigeorgiou AG (2016). Analyzing MiRNA-LncRNA interactions. Methods in Molecular Biology.

[19] Petrica L, Vlad A, Gluhovschi G, Gadalean F, Dumitrascu V, Vlad D (2015). Glycated peptides are associated with the endothelial variability in the kidney and cerebral vessels in type 2 diabetes mellitus patients. J Diabetes Complications.

[20] Petrica Ligia, Sorin Ursoniu, Florica Gadalean, Adrian Vlad, Gheorghe Gluhovschi, Victor Dumitrascu (2017). Urinary podocyte-associated mRNA levels correlate with proximal tubule dysfunction in early diabetic nephropathy of type 2 diabetes mellitus. Diabetology and Metabolic Syndrome.

[21] KDIGO Clinical Practice Guideline for the Evaluation and Management of Chronic Kidney Disease Kidney Int. 2013; 3(Suppl): 1-150.

[22] Schmittgen TD, Livak KJ (2008). Analyzing real-time PCR data by the comparative CT method. Nature Protocols.

[23] Zhou X, Liu S, Cai G, Kong L, Zhang T, Ren Y (2015). Long noncoding RNA MALAT1 promotes tumor growth and metastasis by inducing epithelial-mesenchymal transition in oral squamous cell carcinoma. Scientific Reports.

[24] Yan B, Tao ZF, Li XM, Zhang H, Yao J, Jiang Q (2014). Aberrant expression of long noncoding RNAs in early diabetic retinopathy. Investigative Ophthalmology and Visual Science.

[25] Puthanveetil P, Chen S, Feng B, Gautam A, Chakrabarti S (2015). Long Non-coding RNA MALAT1 regulates hyperglycaemia induced inflammatory process in the endothelial cells. J Cell Mol Me.

[26] Liu JY, Yao J, Li XM, Song YC, Wang XQ, Li YJ (2014). Pathogenic role of lncRNA-MALAT1 in endothelial cell dysfunction in diabetes mellitus. Cell Death Dis.

[27] Li X, Zeng L, Cao C, Lu C, Lian W, Han J (2017). Long noncoding RNA MALAT1 regulates renal tubular epithelial pyroptosis by modulated miR-23c targeting of ELAVL1 in diabetic nephropathy. Exp Cell Res.

[28] Thomson DW, Dinger ME (2016). Endogenous microRNA sponges: evidence and controversy. Nat Rev Genet.

[29] Zhong X, Chung AC, Chen HY, Dong Y, Meng XM, Li R (2013). MiR-21 is a key therapeutic target for renal injury in a mouse model of type 2 diabetes. Diabetologia.

[30] Long J, Wang Y, Wang W, Chang B, Danesh F (2010). Identification of microRNA-93 as a novel regulator of vascular endothelial growth factor in hyperglycemic conditions. J Biol Chem.

[31] Yang J, Shen Y, Yang X, Long Y, Chen S, Lin X (2019). Silencing of long noncoding RNA XIST protects against renal interstitial fibrosis in diabetic nephropathy via microRNA-93-5p-mediated inhibition of CDKN1A. Am J Physiol Renal Physiol.

[32] Qin W, Chung A, Huang X, Meng XM, Hui D, Yu CM (2011). TGF-β/Smad3 signaling promotes renal fibrosis by inhibiting miR-29. J Am Soc Nephrol.

[33] Sun SF, Tang PHK, Feng M, Xiao J, Huang XR, Li P (2018). Novel lncRNA Erbb4-IR promotes diabetic kidney injury in db/db mice by targeting miR-29b. Diabetes.

[34] Chen HY, Zhong X, Huang XR, Meng XM, You Y, Chung ACK (2014). MicroRNA-29b inhibits diabetic nephropathy in db/db mice. Mol Ther.

[35] Lin CL, Lee PH, Hsu YC, Lei CC, Ko JY, Chuang, P C (2014). MicroRNA-29a promotion of nephrin acetylation ameliorates hyperglycemia-induced podocyte dysfunction. J Am Soc Nephrol.

[36] Wang X, Xu Y, Zhu YC, Wang YK, Li J, Li XY (2019). LncRNA NEAT1 promotes extracellular matrix accumulation and epithelial-to-mesenchymal transition by targeting miR-27b-3p and ZEB1 in diabetic nephropathy. J Cell Physiol.

[37] Huang S, Xu Y, Ge X, Xu B, Peng W, Jiang X (2019). Long noncoding RNA NEAT1 accelerates the proliferation and fibrosis in diabetic nephropathy through activating Akt/mTOR signaling pathway. J Cell Physiol.

[38] Li N, Jia T, Li YR (2020). LncRNA NEAT1 accelerates the occurrence and development of diabetic nephropathy by sponging miR-23c. Eur Rev Med Pharmacol Sci.

[39] Leti F, DiStefano JK (2017). Long Noncoding RNAs as diagnostic and therapeutic targets in type 2 diabetes and related complications. Genes (Basel).

[40] He X, Ou C, Xiao Y, Han Q, Li H, Zhou S (2017). LncRNAs: key players and novel insights into diabetes mellitus. Oncotarget.

[41] Raut SK, Khullar M (2018). The big entity of new RNA world: Long non-coding RNAs in microvascular complications of diabetes. Front Endocrinol (Lausanne).

[42] Sun C, Huang L, Li Z, Leng K, Xu Y, Jiang X (2018). Long non-coding RNA MIAT in development and disease: a new player in an old game. J Biomed Sci.

[43] Hock MB, Kralli A (2009). Transcriptional control of mithochondrial biogenesis and function. Annu Rev Pathol.

[44] Long J, Badal SS, Ye Z, Wang Y, Ayanga BA, Galvan DL (2016). Long noncoding RNA Tug1 regulates mitochondrial bioenergetics in diabetic nephropathy. J Clin Invest.

[45] Wang F, Gao X, Zhang R, Zhao P, Sun Y, Li C (2019). LncRNA TUG1 ameliorates diabetic nephropathy by inhibiting miR-21 to promote TIMP3-expression. Int J Clin Exp Pathol.

